# Validation of KASP markers associated with cassava mosaic disease resistance, storage root dry matter and provitamin A carotenoid contents in Ugandan cassava germplasm

**DOI:** 10.3389/fpls.2022.1017275

**Published:** 2022-11-23

**Authors:** Williams Esuma, Oscar Eyoo, Francisca Gwandu, Settumba Mukasa, Titus Alicai, Alfred Ozimati, Ephraim Nuwamanya, Ismail Rabbi, Robert Kawuki

**Affiliations:** ^1^ National Crops Resources Research Institute, Kampala, Uganda; ^2^ College of Natural Sciences, Department of Plant Sciences, Microbiology and Biotechnology, Makerere University, Kampala, Uganda; ^3^ International Institute of Tropical Agriculture (IITA), Oyo, Nigeria

**Keywords:** allele-specific PCR assay, genetic gain, *Manihot esculenta*, marker-assisted breeding (MAB), vitamin A deficiency

## Abstract

**Introduction:**

The intrinsic high heterozygosity of cassava makes conventional breeding ineffective for rapid genetic improvement. However, recent advances in next generation sequencing technologies have enabled the use of high-density markers for genome-wide association studies, aimed at identifying single nucleotide polymorphisms (SNPs) linked to major traits such as cassava mosaic disease (CMD) resistance, dry matter content (DMC) and total carotenoids content (TCC). A number of these trait-linked SNPs have been converted to Kompetitive allele-specific polymerase chain reaction (KASP) markers for downstream application of marker assisted selection.

**Methods:**

We assayed 13 KASP markers to evaluate their effectiveness in selecting for CMD, DMC and TCC in 1,677 diverse cassava genotypes representing two independent breeding populations in Uganda.

**Results:**

Five KASP markers had significant co-segregation with phenotypes; CMD resistance (2), DMC (1) and TCC (2), with each marker accounting for at least 30% of the phenotypic variation. Markers located within the chromosomal regions for which strong marker-trait association loci have been characterised (chromosome 12 markers for CMD, chromosome 1 markers for DMC and TCC) had consistently superior ability to discriminate the respective phenotypes.

**Discussion:**

The results indicate varying discriminatory abilities of the KASP markers assayed and the need for their context-based use for MAS, with PSY2_572 particularly effective in selecting for high TCC. Availing the effective KASP markers on cost-effective genotyping platforms could facilitate practical implementation of marker-assisted cassava breeding for accelerated genetic gains for CMD, DMC and provitamin A carotenoids.

## Introduction

Cassava (*Manihot esculenta* Crantz), a climate-resilient crop grown on approximately 18 million hectares in Africa, offers great potential to end extreme hunger, achieve food security, improve nutrition and eradicate poverty if ideal varieties are deployed (Kolawole et al., 2010). Cassava’s prominence in Africa’s subsistence farming systems is attributed primarily to the crop’s competitive advantage to produce reasonable yields under adverse environments where other crops would fail (Ogola and Mathews, 2011) or where resource-poor farmers cannot afford modern inputs required for intensive farming. The crop is mainly cultivated by smallholder farmers for its starchy roots, which are consumed when boiled or processed into products such as flour for preparing meals (Iragaba et al., 2020).

However, the inherent heterozygous nature, long breeding cycles and high sensitivity to environmental variations make conventional cassava breeding a difficult and expensive task (Ceballos et al., 2015). Integrating breeding innovations such as marker-assisted selection (MAS) into cassava improvement programs is expected to increase the efficiency and speed of variety development (Ferguson et al., 2011). Indeed, marker-assisted introgression of cassava mosaic disease (CMD) resistance into Latin American cassava germplasm prior to its introduction to Africa was a pioneer success story of classical MAS (Okogbenin et al., 2012).

Application of MAS in cassava breeding programs would help in several ways, including: (i) elimination of genotypes with unfavorable alleles at the early stage of the selection scheme, thus allowing for field testing of a reduced number of genotypes, (ii) rapid introgression of resistant genes into existing cassava clones, in places where the disease has recently spread to, for example in Southeast Asian countries (Uke et al., 2022), (iii) early selection of traits that are best measured at the late crop developmental stages, (iv) selection for resistance under environments where the disease pressure is low; and (v) discrimination of genotypes homozygosity and/or heterozygosity. Despite the promise from MAS, its use in cassava breeding is limited and lags considerably behind progress in other major crops such as maize, rice and wheat. An important technical impediment in deploying markers in cassava breeding is the failure to translate genomic knowledge into user-friendly assays that are robust enough to support selection decisions (Ferguson et al., 2011).

The advent of next generation sequencing technologies has renewed the hope for MAS in plant breeding by enabling the identification and use of trait-linked single nucleotide polymorphism (SNP) markers in selection. With sequencing and annotation of the cassava genome nearly complete (Prochnik et al., 2012), it is now possible to use genome-wide markers (Elshire et al., 2011) in genome-wide association studies (GWAS) for identification of trait-linked genomic regions and precisely anchor SNPs at such loci (Oliveira et al., 2012). Recent studies have used genome-wide mapping to identify SNPs associated with provitamin A carotenoids content (Esuma et al., 2016), cassava brown streak diseases resistance (Kayondo et al., 2018), CMD resistance (Rabbi et al., 2014), green mite resistance (Ezenwaka et al., 2018) and dry matter content (Rabbi et al., 2022). To facilitate their use for MAS in cassava breeding, some of the trait-linked SNPs identified by Rabbi et al. (2022) have been converted into Kompetitive allele-specific polymerase chain reaction (KASP) assays (Ige et al., 2021). KASP markers have three major advantages over other molecular marker techniques: low cost, high throughput, and high specificity and sensitivity. These attributes have already made KASP markers a popular choice for MAS in crops such as rice (Sandhu et al., 2022), wheat (Grewal et al., 2022) and soybean (Rosso et al., 2021).

In the case of cassava, the use of KASP markers in selection decisions is yet to be fully integrated into breeding programs. Effective deployment of markers for routine MAS requires an assessment of their predictive ability through technical and biological validations in independent populations (Chagné et al., 2019 ). Technical validation would provide information on the marker call rate and clarity of genotype classes, while biological validation pinpoints the ability of markers to predict a phenotype. This study was undertaken to evaluate the effectiveness of selected KASP markers CMD resistance, dry matter content (DMC) and total carotenoids content (TCC) in Uganda’s cassava breeding population.

## Materials and methods

### Germplasm and phenotyping

Two diverse cassava populations from Uganda’s breeding program at National Crops Resources Research Institute (NaCRRI) were used as independent genetic resource for the KASP marker validation. One population was constituted by 653 genotypes segregating for DMC and TCC in the provitamin A cassava breeding pipeline (pVAC population) while the second trial had 1,024 genotypes (white-fleshed population) segregating for CMD resistance. The two populations were phenotyped concurrently at Namulonge, central Uganda, during 2018/2019 cropping season. Namulonge is a known hotspot for CMD due to the high prevalence of cassava geminiviruses and super abundance of whitefly vector population in the area. Each trial was laid down in an augmented design using 10 plants per plot and two checks replicated within each block, all planted at spacing of 1 x 1 m. Furthermore, rows of CMD infected plants of cultivar BAO (highly susceptible to CMD) were planted as source of inoculum within and around the trial for white-fleshed population to increase disease pressure. Plants were allowed to grow in field under natural conditions for 12 months, with weeding done regularly when needed.

CMD severity was scored on plant basis at three, six and nine months after planting using the 1-5 scale (IITA, 1990), where 1 = no observable symptoms; 2 = mild chlorotic pattern on entire leaflets, mild distortion on the leaves; 3 = pronounced mosaic pattern on the entire leaf, narrowing and distortion of the lower one third of the leaflets; 4 = severe mosaic pattern, distortion of two thirds of leaflets and general reduction of leaf size and stunting plants; 5 = very severe mosaic pattern, distortion of four fifths or more of leaflets, twisted and severe reduction of leaf size in most leaves and severe stunting of plants. At 12 months, plants were uprooted for assessment of TCC and DMC in roots. TCC was assessed by visually scoring the intensity of pigmentation of the root parenchyma on a qualitative scale of 1-8 (Chávez et al., 2005). We used the visual color scale as a high throughput measurement of TCC, aware that previous reports have indicated strong positive correlation between carotenoid content assessed visually and quantitatively (Esuma et al., 2016). To estimate DMC, approximately 200 g of fresh root samples were dried in oven to a constant weight at 105 °C for 24 hours. DMC was then computed as:


$$\text{DMC}\left(\%\right)=\frac{\text{DSW}}{\text{FSW}}\times 100$$

where DSW = dry sample weight and FSW = fresh sample weight.

### KASP marker genotyping

Thirteen KASP markers associated with CMD (3), DMC (4) and TCC (6) were selected from genomic resource under the Next Generation Cassava Breeding project (https://www.nextgencassava.org/). The markers were a product of GWAS using a West African cassava germplasm (Rabbi et al., 2022) from which SNPs with significant marker-trait association were converted to KASP markers at Intertek Laboratory, Australia, as a central repository for coordinated genotyping service. Specific details of the conversion and validation of trait-linked SNPs into uniplex KASP genotyping assays is provided by Ige et al. (2021). **Table 1** presents some statistical and genomic profiles of markers tested.

**Table 1:** Summary statistics of 13 KASP markers validated for CMD, DMC and TCC.

Trait	Marker	Intertek ID	Chr	Minor allele	Major allele	β	SE	p-value
CMD	S12_7926132	snpME0021	12	G	T*	0.89	0.02	p≈0
CMD	S12_7926163	snpME0022	12	A	G*	0.89	0.02	p≈0
CMD	S14_4626854	snpME0025	14	A*	G	-0.23	0.03	1.0x10^-14^
DMC	S1_24197219	snpME0027	1	T	C*	0.77	0.04	p≈0
DMC	S6_20589894	snpME0038	6	G*	A	0.78	0.09	1.7x10^-16^
DMC	S12_5524524	snpME0040	12	C*	T	0.69	0.01	8.0x10^-12^
DMC	S15_1012346	snpME0029	15	C	T*	-0.84	0.01	4.0x10^-17^
TCC	PSY2_572	snpME0001	1	A*	C	0.37	0.01	1.3x10^-219^
TCC	S1_24636113	snpME0043	1	G*	A	0.57	0.02	1.3x10^-270^
TCC	S1_30543962	snpME0047	1	G*	A	0.40	0.02	2.4x10^-76^
TCC	S5_3387558	snpME0053	5	T*	C	0.20	0.02	2.0x10^-16^
TCC	S8_25598183	snpME0056	8	T*	G	0.18	0.03	8.0x10^-12^
TCC	S15_7659426	snpME0049	15	G	T*	-0.06	0.01	4.0x10^-17^

CMD, cassava mosaic disease; DMC, dry matter content; TCC, total carotenoid content; β, SNP effect from associated GWAS; SE, standard error; p-value, probability value for marker-trait association; *Favorable allele. Marker information extracted from Rabbi et al. (2022) and Ige et al. (2021).

Tissues were collected from young newly expanded leaves of plants growing under natural field conditions. Four leaf discs of 6 mm diameter were punched into wells of sample collection plates and desiccated using silica gel for 48 hours. Plates containing dry leaf tissues were shipped to Intertek, Australia. The KASP marker details are available through the Excellence in Breeding repository here: https://excellenceinbreeding.org/module3/kasp; individual marker IDs are further provided in **Table 1**. Details of the procedure for preparation and running of KASP reactions are provided in the KASP manual (available online: https://www.biosearchtech.com/). Briefly, the genotyping used the high throughput PCR SNPline workflow using 1 μL reaction volume in 96-well plates for PCR. The KASP genotyping reaction mix comprised three components: (i) sample DNA (~10 ng); (ii) marker assay mix consisting of target-specific primers; and (iii) KASP-TFTM Master Mix containing two universal FRET (fluorescence resonant energy transfer) cassettes (FAM and HEX), passive reference dye (ROX™), Taq polymerase, free nucleotides, and MgCl_2_ in an optimized buffer solution. The SNP assay mix was specific to each marker and consisted of two Kompetitive allele-specific forward primers and one common reverse primer. Finally, the PCR products were fluorescently read, and allele calls made using KRAKENTM software.

### Data analysis

#### Phenotypic data analysis

Because some genotypes infected with CMD tend to recover during the plant growing stage, we used CMDs scored at nine months as the optimal data for subsequent analyses. Phenotypic data for each trial were considered independent and fitted separately into linear mixed models with the *lme4* package for R statistical software (Bates et al., 2015) to allow for extraction of best linear unbiased predictions (BLUPs) of the genotype effects for CMD, TCC and DMC. In each case, we fitted the following linear mixed model:


$$\text{y}=\text{X}\beta +{\text{Z}}_{{\text{g}}^{c}}+{\text{Z}}_{{\text{block}}^{\text{b}}}+\epsilon$$

Where *y* was the vector of raw phenotype, β was a fixed effect of grand mean with the corresponding incidence matrix *X*, vector *c* and corresponding incidence matrix *Z_g_* was the random effect for genotypes (*g*) such that $\;c∼\text{N}\left(0,\text{I}\sigma_{e}^{2}\right)$ , *Z*_*block* ^*B*^_ represented the random effect for blocks and ϵ was the residual such that $e∼\text{N}\left(0,\text{I}\sigma_{e}^{2}\right)$ . In the model, checks were considered as fixed effects while accessions and blocks were considered random effects. Variance components were extracted from the models for estimation plot-based heritability (*H^2^*) as:


$${\text{H}}^{2}=\frac{\sigma_{c}^{2}}{\left(\sigma_{c}^{2}+\sigma_{e}^{2}\right)}$$

where $\sigma_{c}^{2}$ was the genotype variance and $\sigma_{e}^{2}$ was the model residual variance. BLUPs for each genotype were extracted using the *ranef* function in *lme4* package.

### Marker segregation and marker effects

Boxplots drawn with the *ggpubr* package in R were used to visualize segregation of marker genotypes for each phenotype, and statistical differences among the genotypes were compared using the Kruskal-Wallis test. Marker effects were further evaluated by regressing the marker genotypes onto respective phenotypes for estimation of phenotypic variance accounted to by each marker. In this case, linear regression was performed using the *lm* function in R such that marker genotypes and the corresponding phenotypes were treated as independent and response variables, respectively.

### Estimation of biological metrics for CMD markers

The KASP markers for CMD resistance targeted the CMD2 locus (Rabbi et al., 2014; Wolfe et al., 2016), which has been classified as dominant monogenic trait (Okogbenin et al., 2012) and is expected to segregate in Mendelian fashion. Therefore, we used confusion matrix to estimate some performance statistics to determine the ability of each CMD KASP marker in predicting the response of genotypes for CMD resistance or susceptibility. The performance statistics included: a) accuracy (ACC), which is the proportion of correctly predicted genotypes, either as resistant or susceptible; b) false positive rate (FPR), which is the proportion of the genotypes predicted to be resistant but were diseased (also referred to as type I error); and c) the false-negative rate (FNR) which is the proportion of genotypes predicted to be susceptible but were resistant (type II error). These statistics were computed as:


$$\text{ACC}=\frac{\text{TP}+\text{TN}}{\text{ TP}+\text{FP}+\text{TN}+\text{FN}}$$

$$\text{FPR}=\frac{\text{FP}}{\text{FP}+\text{TN}}$$

$$\text{FNR}=\frac{\text{FN}}{\text{FN}+\text{TP}}$$

Where FP *=* false positive, TN = true negative, FN = false negative, TP *=* true positive (Ige et al., 2021).

## Results

### Phenotypic variation and broad-sense heritability for CMD, DMC and TCC

**Figure 1:**
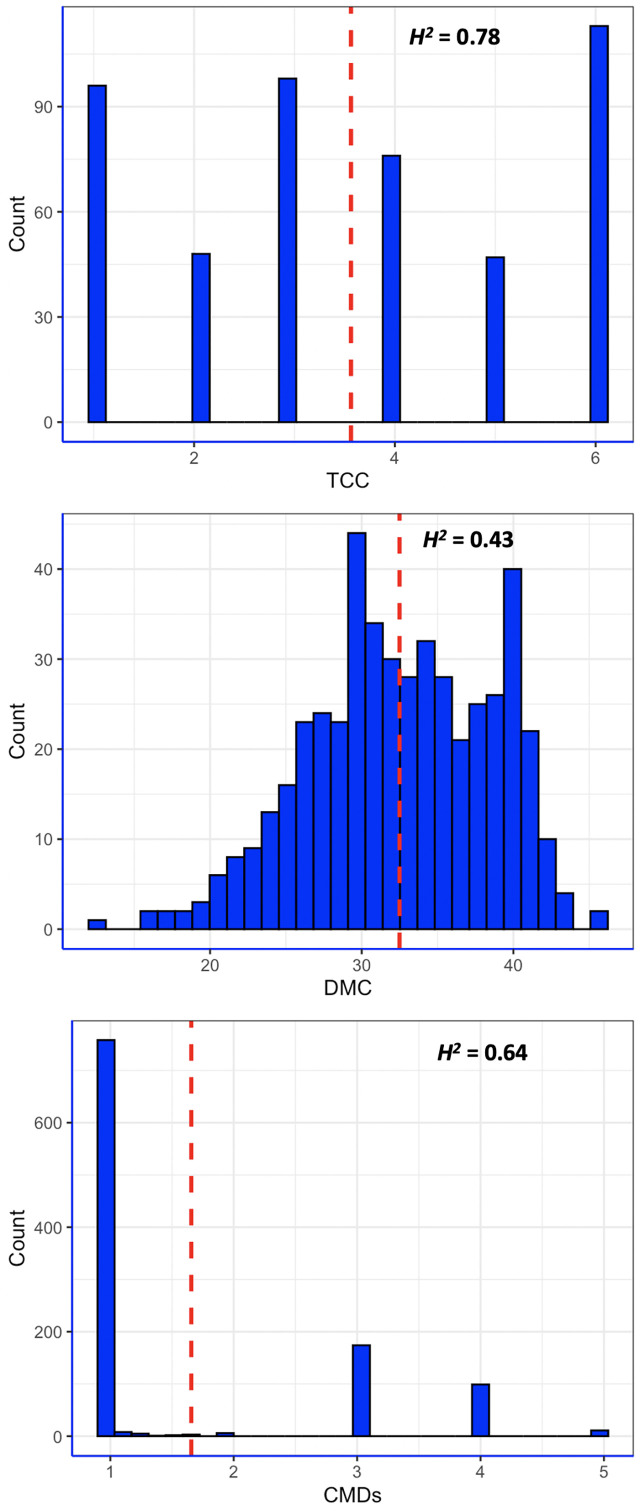
Distribution of phenotypic values of three traits measured in this study. CMDs = severity of cassava mosaic disease; DMC = dry matter content; TCC = total carotenoids content; *H^2^* = broad-sense heritability; red dashed line is the mean value for each trait.

All the three traits phenotyped had considerable variation, with CMDs showing typical bimodal distribution that had clear-cut separation between symptomless plants and those with varying severity levels (**Figure 1**). The white-fleshed population evaluated for CMD had 73.4% of the genotypes scoring 1 or 2 for CMD severity and were subsequently categorized as resistant (Lim et al., 2022), while the remaining genotypes were susceptible, with CMDs varying between 3 and 5. The mean CMDs score in the population was 1.67. In contrast, DMC and TCC exhibited continuous distribution pattern, akin to quantitative traits. DMC varied between 12.3% and 45.4%, with an average of 31.5%. Root pigmentation, a qualitative measure of TCC, varied from 1 (white) to 6 (deep yellow), with a mean of 4.0. In fact, 85.1% of the pVAC population had TCC score varying between 2 and 6. Broad-sense heritability of CMD, DMC and TCC were 0.64, 0.43 and 0.78, respectively (**Figure 1**).

### Allele profiles and frequencies of markers tested

**Figure 2:**
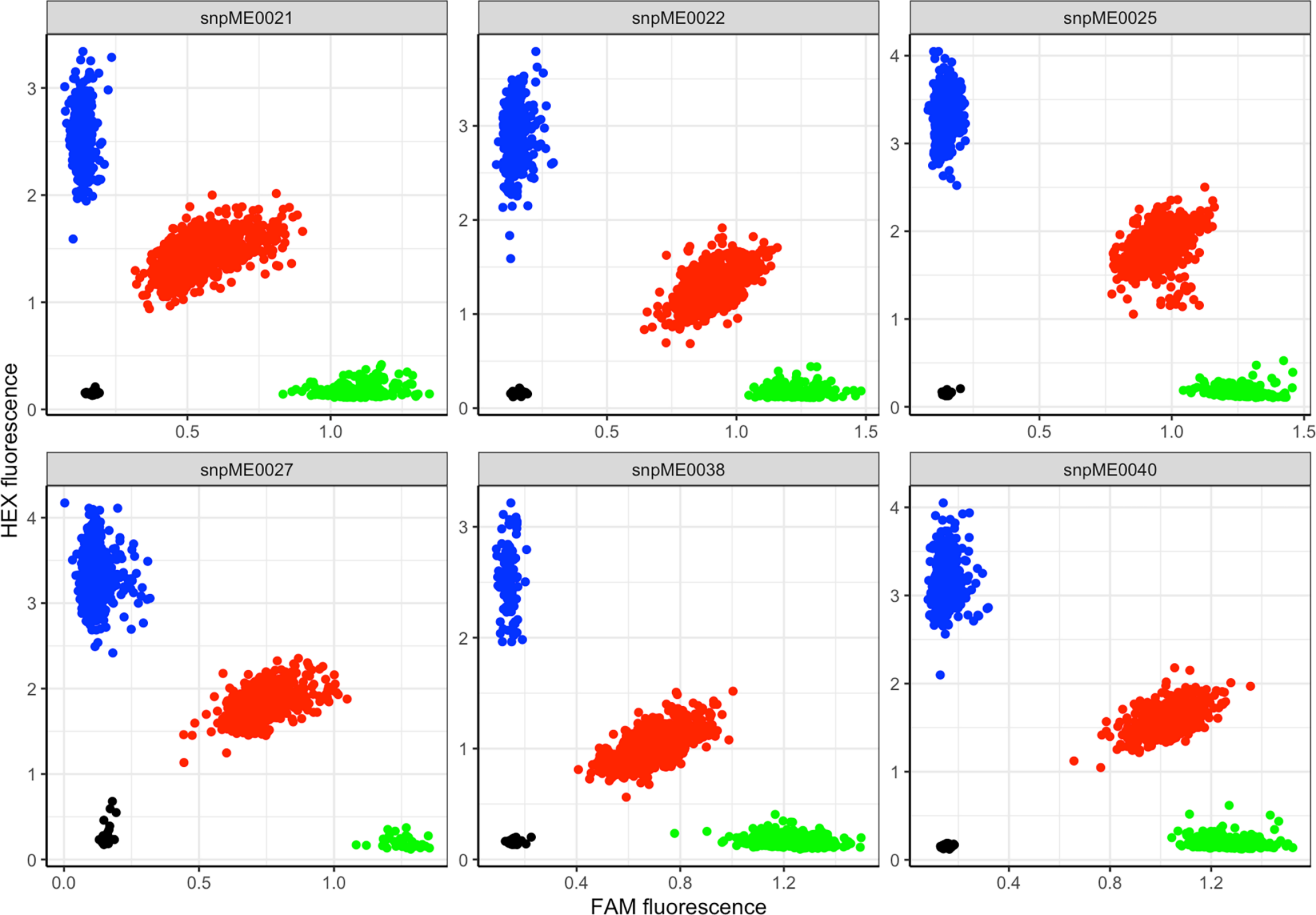
Scatter plot for selected KASP assays showing clustering of genotypes on the *Y*- and *X-*axes. Genotypes colored blue have a HEX-type allele; genotypes colored green have a FAM-type allele; genotypes colored red are heterozygotes; black dots represent non-template controls.

Based on fluorescence profiles of the KASP assays, the allele calls of each marker clustered into three distinct groups: homozygous genotypes with a HEX-type allele, homozygous genotypes with a FAM-type allele and heterozygotes (**Figure 2**). The allelic states corresponded to homozygous genotypes for minor alleles, homozygous genotypes for major alleles and heterozygotes having both alleles. Meanwhile, there was an overall high call rate (>97%) for the 13 KASP markers assayed, except for marker S15_1012346 (DMC) which had a comparably low call rate of 84.5% (**Table 2**). In the case of CMD, all the three markers (S12_7926132, S12_7926163 and S14_4626854) had a consistent pattern of allele distribution, with heterozygotes having the highest frequency (>56%) followed by homozygous genotypes for major alleles. The frequency of the homozygous state of minor alleles for CMD markers varied between 15% (marker S14_4626854) and 20% (markers S12_7926132 and S12_7926163), while the frequency of major alleles ranged from 20% to 29%. Meanwhile, distribution of allele frequencies for DMC and TCC markers did not follow any specific pattern. For example, the homozygous state of minor and major alleles of marker S15_1012346 (DMC) had equal frequency (16.7%) while the heterozygotes were 51.1%. On the contrary, homozygous state of the minor allele for marker S8_25598183 (TCC) had frequency of 70.8%, while the homozygous state of its major allele frequency was 1.1%.

**Table 2:** Frequency of 13 KASP marker genotypes segregating for CMD, DMC and TCC in Uganda’s cassava breeding population.

Trait	Marker	N	Hom1	Het	Hom2	%Hom1	%Het	%Hom2	% Null
CMD	S12_7926132	1,024	GG	TG	TT	19.9	59.3	20.2	0.6
CMD	S12_7926163	1,024	AA	GA	GG	19.9	59.5	20.5	0.1
CMD	S14_4626854	1,024	AA	AG	GG	14.9	56.1	28.7	0.3
DMC	S1_24197219	653	TT	TC	CC	46.5	28.3	7.0	0.2
DMC	S6_20589894	653	GG	GA	AA	30.2	52.5	16.7	0.6
DMC	S12_5524524	653	CC	CT	TT	8.1	49.3	42.1	0.5
DMC	S15_1012346	653	CC	CT	TT	16.7	51.1	16.7	15.5
TCC	PSY2_572	653	AA	CA	CC	61.1	29.9	8.3	0.8
TCC	S1_24636113	653	GG	GA	AA	55.3	32.5	9.8	2.5
TCC	S1_30543962	653	GG	GA	AA	19.9	47.3	30.5	2.3
TCC	S5_3387558	653	TT	TC	CC	0.6	75.8	23.1	0.5
TCC	S8_25598183	653	TT	TG	GG	70.8	27.6	1.1	0.6
TCC	S15_7659426	653	GG	GT	TT	49.3	44.1	6.4	0.2

Hom1, homozygous for minor allele; Het, heterozygote; Hom2, homozygous for major allele; Null, uncallable genotypes.

### Marker effects on traits

**Figure 3:**
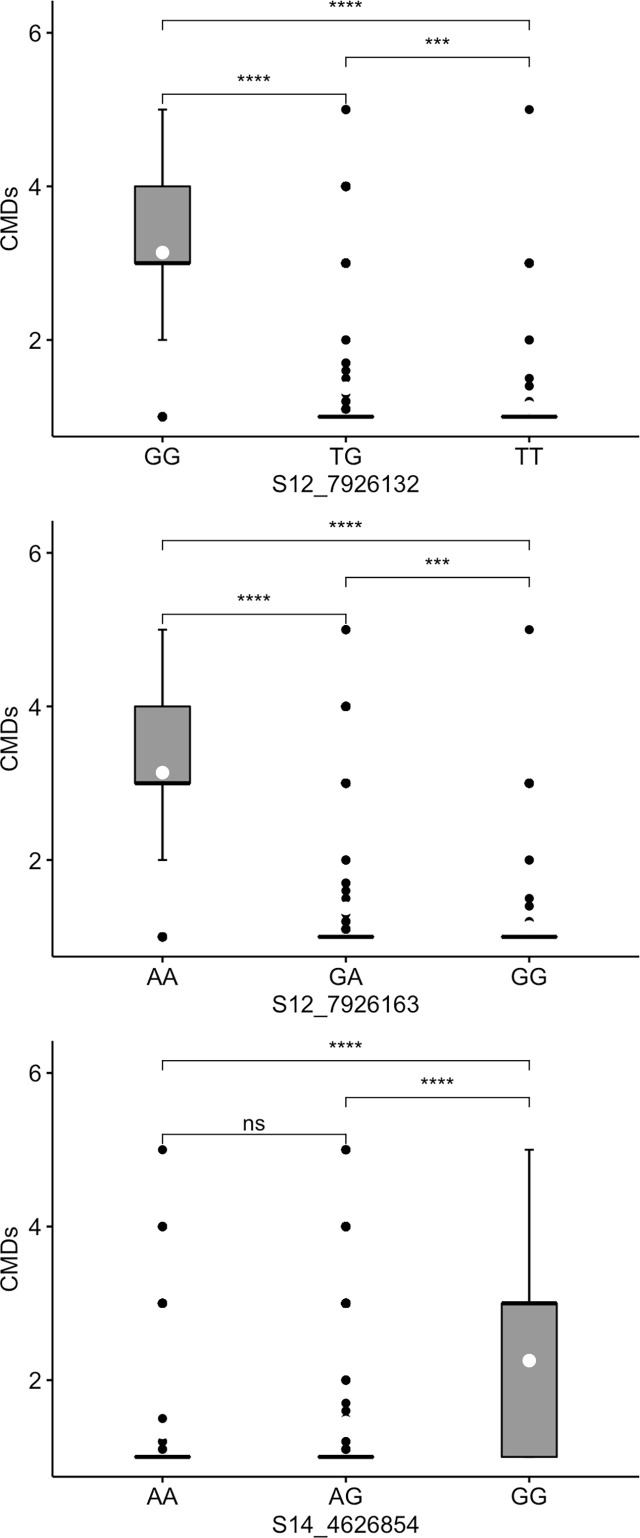
Box plots showing co-segregation of three KASP markers with cassava mosaic disease severity (CMDs). ^****^, ^***^ significant at p ≤ 0.0001, p ≤ 0.001 and p ≤ 0.01, respectively; ^ns^ = non-significant.

Marked differences were observed in the allele substitution effects across all the 13 markers and within markers for each trait. CMD markers S12_7926132 and S12_7926163, which are only 31 bp apart, exhibited segregation patterns typical of dominant markers. For marker S12_7926132, genotypes with at least one copy of the favorable (resistance) allele (TG or TT) significantly co-segregated with low CMD severity compared to the unfavorable allele for which the associated genotypes had mean CMD severity >3 (**Figure 3**). Similarly, genotypes with a copy of the favorable allele G (GA or GG) for marker S12_7926163 showed significant co-segregation with CMD resistant clones. For both markers, the heterozygotes had different performance when compared to that of genotypes homozygous for favorable allele. For marker S14_4626854, genotypes with the favorable allele A (AA or AG) had significantly low scores of CMDs compared to those carrying GG. However, in this marker, the heterozygotes had similar performance to that of genotypes with homozygous favorable allele (AA).

**Figure 4:**
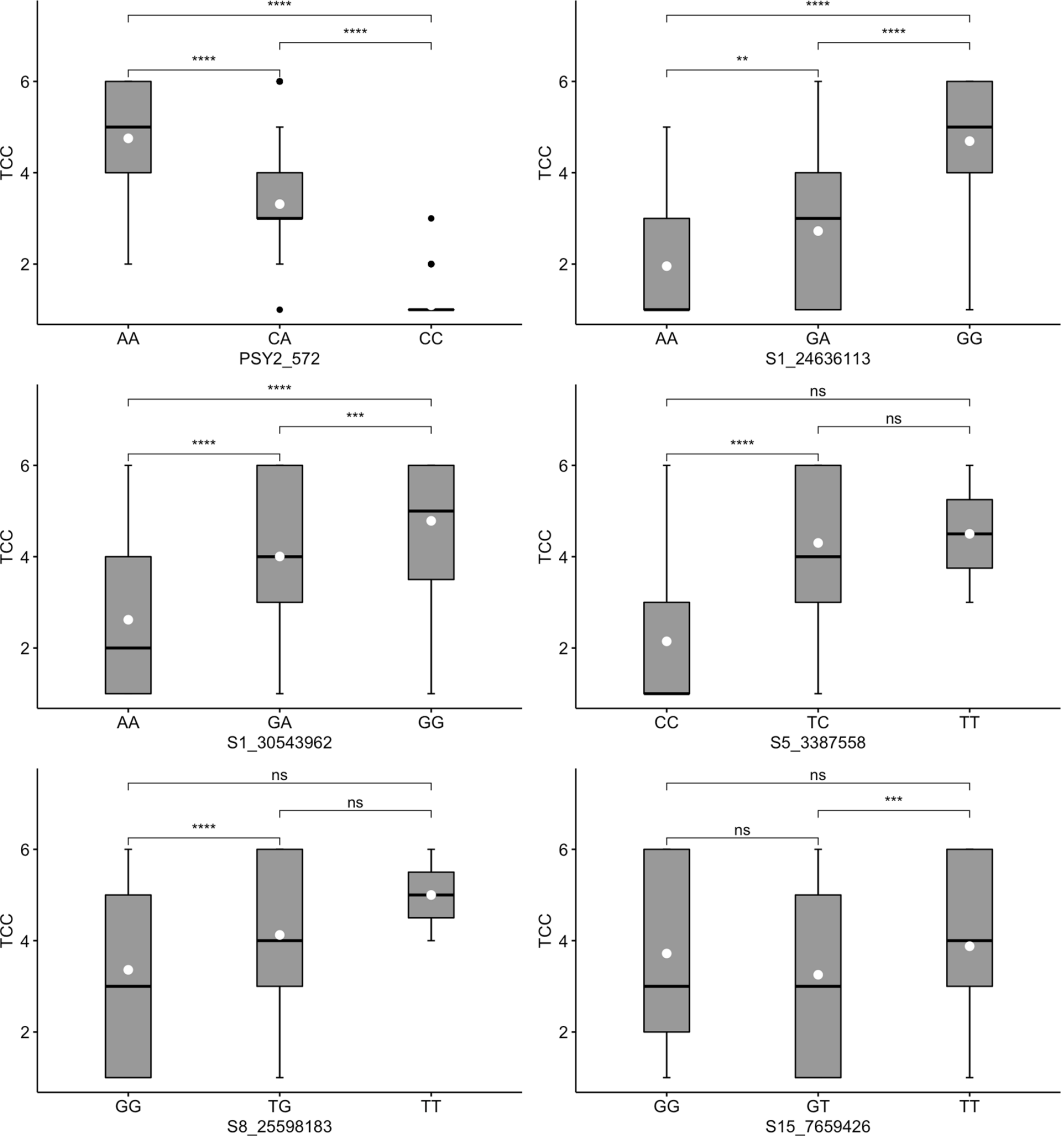
Box plots showing co-segregation of six KASP markers with total carotenoids content (TCC). ^****^, ^***^, ^**^ significant at p ≤ 0.0001, p ≤ 0.001 and p ≤ 0.01, respectively; ^ns^ = non-significant.

Three markers tested for TCC (PSY2_572, S1_24636113 and S1_30543962) also had segregation patterns likened to dominant gene effect. For marker PSY2_572, genotypes with at least one copy of the favorable allele (AA or CA) co-segregated with high levels of TCC (**Figure 4**). Similarly, markers S1_24636113 and S1_30543962 had significantly higher levels of TCC in genotypes carrying favorable alleles in the form of GA/GG and TC/TT, respectively. Evidently, these markers exhibited additive allelic effects, with mean TCC levels consistently higher in genotypes homozygous for favorable alleles than those in heterozygotes. The other three markers evaluated for TCC (S5_3387558, S8_25598183 and S15_7659426) did not show segregation patterns consistent with expression of the trait in genotypes assayed. For example, there were non-significant differences between marker-phenotype co-segregation of the two homozygous allelic states for each of the three markers.

**Figure 5:**
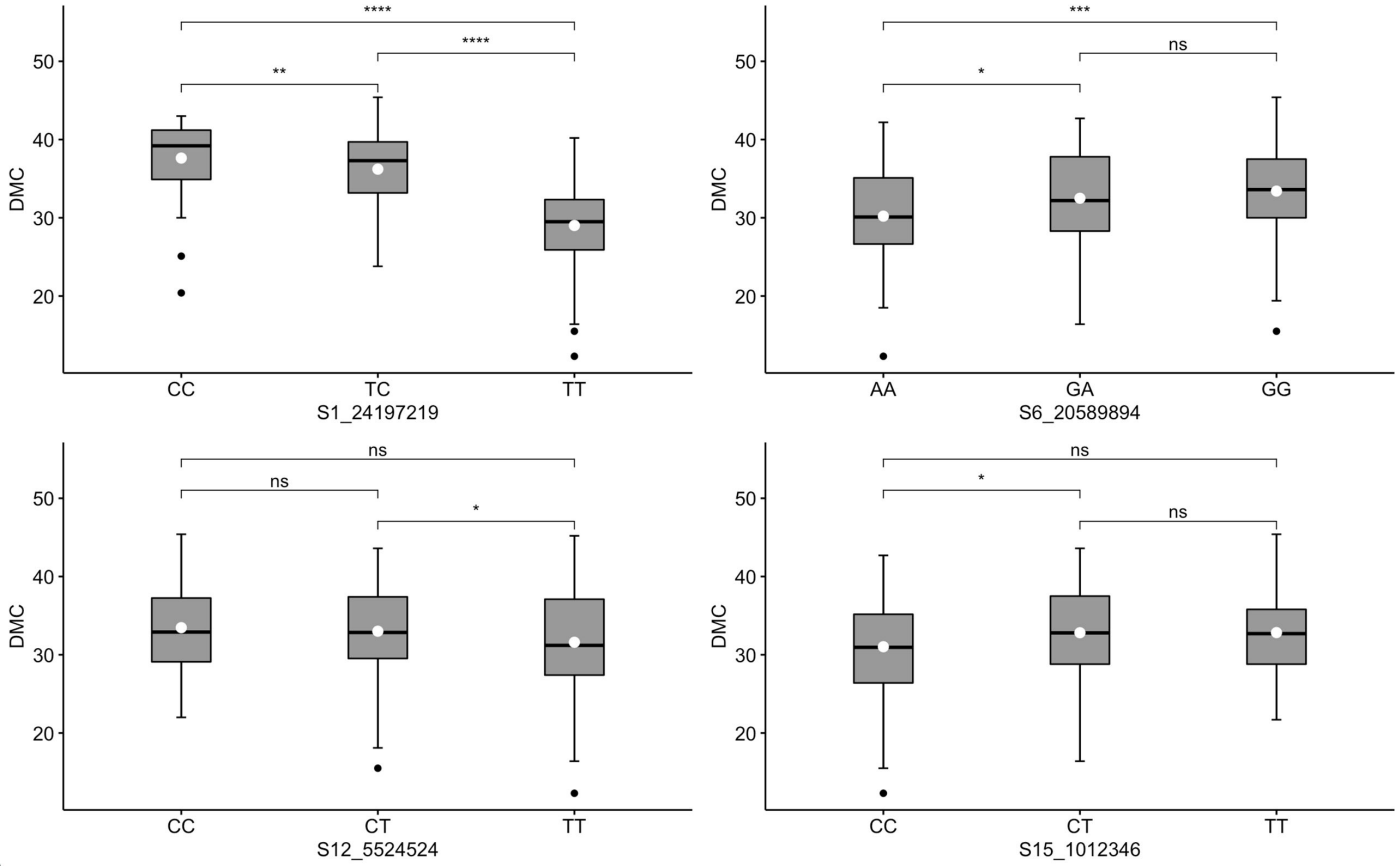
Box plots showing co-segregation of six KASP markers with dry matter content (DMC). ^****^, ^***^, ^**^, ^*^ significant at *p* ≤ 0.0001, *p* ≤ 0.001, *p* ≤ 0.01 and *p* ≤ 0.05, respectively; ^ns^ = non-significant.

For DMC, two markers (S1_24197219 and S6_20589894) exhibited dominant segregation patterns (**Figure 5**). In the case of marker S1_24197219, genotypes CC and TC co-segregated with high DMC. A similar observation was noted for marker S6_20589894, for which genotypes GA and GG were significantly associated with high values of DMC. Although not pronounced, these two markers manifested additivity, with genotypes carrying homozygous favorable alleles having the highest levels of DMC. Meanwhile, segregation patterns in the other two DMC markers (S12_5524524 and S15_1012346) were inconsistent with the phenotypic distribution, where the two homozygous allelic states of each marker had nonsignificant co-segregation with the trait.

**Figure 6:**
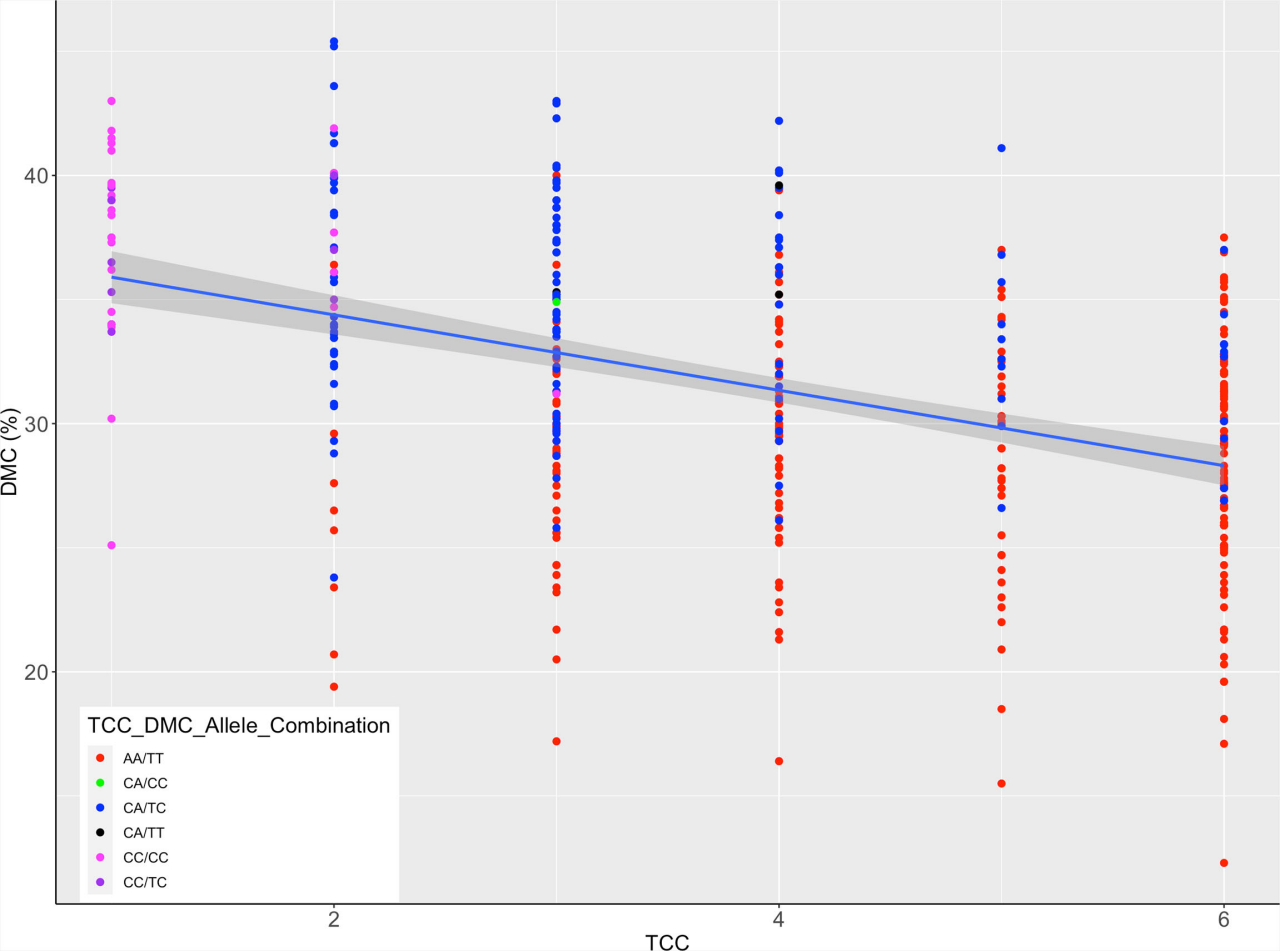
Scatter plot of total carotenoids content (TCC) and dry matter content (DMC) scaled by alleles for markers PSY2_572 and S1_24197219; the legend shows TCC/DMC allelic combinations of the two markers in genotypes assayed; the allelic states correspond to homozygous favorable alleles, heterozygotes and the wild type, as indicated in **Figures 4**, **5**.

An inverse relationship was noted between TCC and DMC (**Figure 6**) for genotypes assayed. When the allelic further profiles of best performing markers for TCC (PSY2_572) and DMC (S1_24197219) were further examined, all genotypes homozygous for the favorable allele for TCC (AA) did not have the favorable allele for DMC (**Figure 6**) and had generally low levels of DMC. Similarly, genotypes homozygous for the favorable allele for DMC (CC) did not have the favorable allele for TCC and had low total carotenoids content, with exception of one genotype scoring CA/CC. However, there were 116 genotypes combining heterozygous states of the two markers (CA/TC).

When we regressed the marker genotypes onto the respective phenotypes, all markers showed significant association with traits, except two markers (S8_25598183 and S15_7659426) for TCC (**Table 3**). However, the proportion of phenotypic variance attributable to the markers was generally low, with the highest value recorded for CMD markers S12_7926132 and S12_7926163 (*R^2^* = 0.45). Only one marker (S1_24197219) accounted for up to 30% of variation in DMC, with the remaining three explaining ≤5% of the phenotypic variance. Meanwhile, markers PSY2_572 and S1_24636113 accounted for the 42% and 30%, respectively. All other TCC markers had low *R^2^* values, with S1_30543962 accounting for 15% of the phenotypic variation and S15_7659426 explaining none of the observable variation in the trait.

**Table 3:** Regression coefficients for 13 KASP markers tested for CMD, DMC and TCC in Ugandan cassava germplasm.

Trait	Marker	df	ms	*R^2^*
CMD	S12_7926132	2	322.8^***^	0.45
CMD	S12_7926163	2	322.6^***^	0.45
CMD	S14_4626854	2	96.6^***^	0.13
DMC	S1_24197219	2	1897.6^***^	0.30
DMC	S6_20589894	2	299.6^***^	0.05
DMC	S12_5524524	2	241.0^***^	0.04
DMC	S15_1012346	2	261.7^***^	0.04
TCC	PSY2_572	2	301.5^***^	0.42
TCC	S1_24636113	2	210.2^***^	0.30
TCC	S1_30543962	2	56.9^***^	0.15
TCC	S5_3387558	2	91.5^***^	0.06
TCC	S8_25598183	2	3.9	0.01
TCC	S15_7659426	2	2.7	0.00

CMD, cassava mosaic disease; DMC, dry matter content; TCC, total carotenoid content; df, degree of freedom; ms, mean square; R^2^, coefficient of determination and; ^***^, significance at p ≤ 0.001.

Principal component analysis further revealed similarities between markers located within the same chromosomal positions. For example, CMD markers S12_7926132 and S12_7926163 had identical contribution to the total variation in the principal components; the same observation was true for TCC markers PSY2_572, S1_24636113 (**Supplementary Figure 1**). Such markers would be considered redundant to each other.

### Predictive ability of CMD markers based on biological metrics

We used biological metrics to further investigate the predictive ability of CMD markers, given qualitative nature of the trait. In this case, markers S12_7926132 and S12_7926163 had comparable predictive abilities. For example, both markers had high prediction accuracy (86.7%) and relatively low false positive rate (~38%) (**Table 4**). In contrast, marker S14_4626854 had somewhat low prediction accuracy (74.5%) and a relatively high false positive rate (44.3%).

**Table 4:** Predictive ability of three KASP markers for CMD in Uganda’s cassava breeding population.

Marker	Prediction	True phenotype		Accuracy (%)	FPR (%)	FNR (%)
		R	S			
S12_7926132	R	699	114	86.7	38.4	2.9
	S	21	183			
S12_7926163	R	703	115	86.7	38.7	2.9
	S	21	182			
S14_4626854	R	594	132	74.5	44.3	17.7
	S	128	166			

R, Resistant; S, Susceptible; FPR, false positive rate; FNR, false negative rate.

## Discussion

The global agricultural production is currently facing unprecedented challenges imposed by rapid human population growth, limited arable land and adverse effects of climate change. These challenges call for greater efforts to optimize and deploy appropriate tools, technologies and methods that can accelerate genetic gains from breeding programs for rapid delivery of high-capacity varieties to farmers. Advances in sequencing technologies can now allow for cost-effective use of genome-wide markers (Elshire et al., 2011) for identification of and use of trait-linked DNA polymorphisms. In the case of cassava, various research investments, including those through the Next Generation Cassava Breeding project (https://www.nextgencassava.org/) have yielded important genomic resources, such as well annotated genome sequence (Prochnik et al., 2012) and collation SNP markers for various agronomic and quality traits (Rabbi et al., 2022). Thus, this study tested the effectiveness of selected trait-linked KASP markers identified from West African gene pool for MAS in Uganda’s cassava breeding population as an independent validation set. By deploying trait-linked markers for MAS, cassava breeders could efficiently select genotypes with desired trait combinations at seedling stage so that inferior individuals for a specific trait are quickly discarded. With a reduced number of clones in subsequent selection stages, breeders can then shift to index selection where multiple traits are improved simultaneously, thereby increasing the speed of variety development and reducing cost of breeding operations (Ceballos et al., 2015).

The cassava improvement program in Uganda implements a demand-led breeding, in which a stage-gate product development is inspired by needs of two main market segments: food (boiled roots and flour) and industry. Dry matter content, provitamin A carotenoids content and virus disease resistance are must-have traits for cassava product profile in Uganda (Iragaba et al., 2020; Esuma et al., 2021). The two cassava breeding populations used in this study have three important attributes that would warrant the use of MAS for rapid improvement of these traits. Firstly, the wide phenotypic variability for CMD, DMC and TCC justifies the use of markers that can efficiently pinpoint desired genotypes in a large segregating population for forward breeding. In fact, DMC and TCC exhibited substantial degree of quantitative variation for which phenotypic selection alone can be slow and expensive. Secondly, the low broad-sense heritability of DMC implies the need for robust field phenotyping before accurate decisions can be taken on a genotype’s genetic merit for the trait. Thirdly, all the three traits can only be optimally estimated on physiologically mature (≥ months old) plants.

The robust and high call rates for the marker genotypes demonstrated their usefulness in screening cassava germplasm of broad genetic background. However, the wide variability in the predictive ability of the markers suggests the need for their case-by-case deployment in cassava breeding. For example, the consistent and significant co-segregation of allelic states of markers S12_7926132 and S12_7926163 with CMD severity indicates their reliability for MAS. In fact, markers S12_7926132 and S12_7926163 may be tightly linked to the functional gene in conferring CMD resistance in cassava given their close proximity to CMD2 resistance locus previously identified on chromosome 12 (Okogbenin et al., 2012; Wolfe et al., 2016; Rabbi et al., 2022). Given, the short distance separating markers S12_7926132 and S12_7926163, using either of them would be sufficient for implementing MAS for CMD2 resistance.

The allelic segregation of markers S12_7926132 and S12_7926163 points to two important aspects. Foremost, the consistent dominant allelic effects exhibited in the marker segregation underpin CMD2 is a qualitative trait under additive genetic effect. Despite the classical qualitative segregation exhibited for CMDs, there was a considerable variation in severity levels for susceptible clones, which could be attributed to differences in plot-based viral loads or other genetic factors controlling plant fitness. Secondly, the large proportion of variation in CMDs unexplained by the marker effects indicates the need for continuous improvement for effective operationalization of MAS platforms in cassava. Through whole genome sequencing and genetic variant analysis, (Lim et al., 2022) fine-mapped the CMD2 locus to a 190 kilobase and identified additional nonsynonymous SNP in *DNA polymerase δ subunit 1 (MePOLD1)* as the functional gene on chromosome 12 responsible for CMD2 resistance. That study generated eight novel KASP markers that, when incorporate into the existing genomic resource, could reinforce prospects of MAS for CMD2 resistance in cassava breeding.

TCC markers PSY2_572, S1_24636113 and S1_30543962 segregated in typical dominant fashion, with additive effects exhibited for allele substitution. These markers are located in a close proximity of *Manes.01G124200.1*, which is a *phytoene synthase* (*PSY*) gene known to increase accumulation of provitamin A carotenoids in cassava roots (Esuma et al., 2016; Rabbi et al., 2017). A detailed characterization of the *PSY* locus by Welsch et al. (2010) indicated that a SNP in *PSY2-Y-2* gene co-segregated with high carotenoids content in cassava roots and the polymorphism resulted in a single amino acid change in a highly conserved region of the protein which increased catalytic activity in *Escherichia coli*. Indeed, *PSY* has also been reported to encode the expression of threonine, a major substance in the carotenoid biosynthetic pathway, in other plant species such as maize varieties with yellow endosperm (Shumskaya et al., 2012; Shumskaya and Wurtzel, 2013) and golden rice (Paine et al., 2005). Thus, it is likely that markers PSY2_572, S1_24636113 and S1_30543962 are in strong linkage disequilibrium with the functional locus controlling synthesis and/or accumulation of provitamin A carotenoids content in cassava roots, and their use for MAS for TCC would be effective. However, marker S1_30543962 accounted for a low proportion of variation in TCC, indicating its ineffectiveness for MAS when used in isolation.

The continuous variability in DMC was typical of a quantitative trait be controlled by many genes with small effects. Nonetheless, markers S1_24197219 and S6_20589894 showed strong co-segregation with DMC. Rabbi et al. (2017) identified a genomic region on chromosome 1 associated with DMC in cassava. The authors annotated two genes *UDP-glucose pyrophosphorylase* and *sucrose synthase* within the DMC association signal. Both genes are known to be essential in the synthesis of sucrose and polysaccharide (Zabotina et al., 2021). Thus, marker S1_24197219, which accounted for the highest proportion of variation in DMC (30%) in our study, could be tightly linked to the functional genes for the trait on chromosome 1 and would be effective for MAS. Based on the segregation pattern and low *R^2^* values for DMC, other markers would be ineffective for implementing MAS, especially in the genetic material evaluated in this study.

The apparent negative correlation between TCC and DMC is a manifestation of the current challenges baffling breeding efforts aimed at delivering cassava varieties with desired end-user traits. In the case of market segment for boiled cassava, there is a high preference for roots with elevated levels of DMC (Iragaba et al., 2020). The KASP marker-based selection tested in this study shows the prospect for rapid and efficient identification of genotypes combining favorable alleles for TCC and DMC at early selection stages and could aid the implementation a rapid cycle recurrent selection scheme in cassava genetic improvement (Ceballos et al., 2015).

Taken together, data presented in this study highlight some prospects for MAS in cassava, especially for CMD, provitamin A carotenoids and DMC. Our data highlighted five markers with sufficient discriminatory ability for MAS: S12_7926132 and S12_7926163 for CMD, PSY2_572 and S1_24636113 for TCC, and S1_24197219 for DMC. The markers should be the focus for cassava breeding programs targeting immediate application of MAS for genetic improvement of these traits. As more genomic tools and resources get optimized for cassava, marker-assisted breeding will become a reality and greatly help increase genetic gains for important agronomic and quality traits that have been too complex to exploit through conventional breeding methods (Ceballos et al., 2012; Ceballos et al., 2015). In the meantime, efforts should be made to enhance the utility and deployment of these markers across the global cassava community to facilitate rapid delivery of varieties that can contribute to reducing poverty and ending hunger, as desired by the first two sustainable development goals.

## Data availability statement

The datasets presented in this study can be found in online repositories. The names of the repository/repositories and accession number(s) can be found below: https://doi.org/10.6084/m9.figshare.21213605.v2.

## Author contributions

WE conceptualized the study, guided field trials and wrote the original manuscript. OE phenotyped trial for CMD and reviewed manuscript. FG phenotyped trial for DMC and TCC. SM guided field trials and edited manuscript. TA guided field trials and edited manuscript. EN guided laboratory analyses for DMC/TCC and edited the manuscript. AO guided data analyses and reviewed manuscript. IR guided marker data analyses and edited manuscript. RK guided the study conceptualization and provided funds. All authors contributed to the article and approved the submitted version.

## Funding

This work was supported through the Next Generation Cassava Breeding project (www.nextgencassava.org/) funded by the Bill and Melinda Gates Foundation through Cornell University (grant number OPP1048542).

## Acknowledgments

We thank IR (IITA, Ibadan) for providing detailed technical information on the 13 KASP markers.

## Conflict of interest

The authors declare that the research was conducted in the absence of any commercial or financial relationships that could be construed as a potential conflict of interest.

The reviewer XZ is currently organizing a Research Topic with one of the authors RK.

## Publisher’s note

All claims expressed in this article are solely those of the authors and do not necessarily represent those of their affiliated organizations, or those of the publisher, the editors and the reviewers. Any product that may be evaluated in this article, or claim that may be made by its manufacturer, is not guaranteed or endorsed by the publisher.
