# Supplementary material for: Validation of KASP markers associated with cassava mosaic disease resistance, storage root dry matter and provitamin A carotenoid contents in Ugandan cassava germplasm
Source: Front Plant Sci. 2022 Nov 23;13:1017275. doi: 10.3389/fpls.2022.1017275 (PMC9727383; doi:10.3389/fpls.2022.1017275)
Supplement: Supplementary file 1 [file DataSheet_1.pdf]

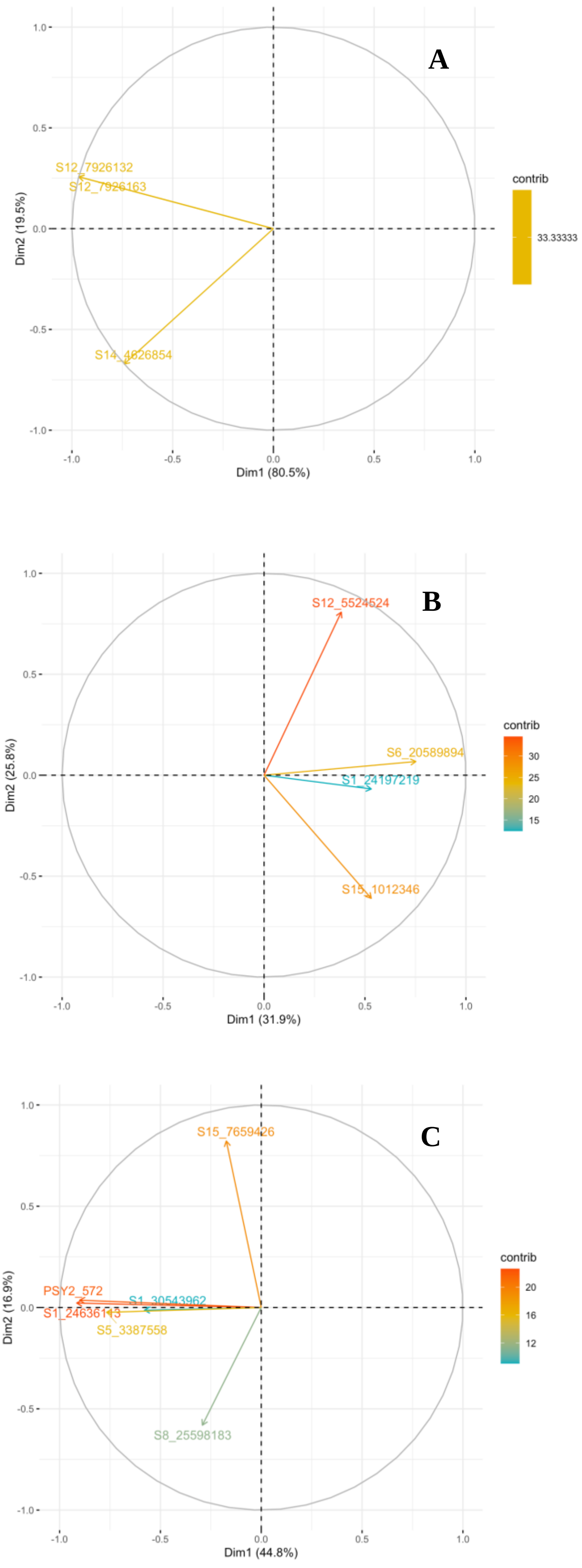

Supplementary Figure 1: Biplots showing relationship among KASP markers for prediction of CMD (A), DMC (B) and TCC (C); Dim1 and Dim2 are the principal components 1 and 2, respectively; the legend (contrib) shows the contribution of each marker to the principal components.
